# Genetic etiology of inherited kidney diseases in egyptian patients: next generation sequencing identifies six novel variants

**DOI:** 10.1186/s40348-025-00203-2

**Published:** 2025-11-25

**Authors:** Nesma M. Elaraby, Ammal M. Metwally, Sara M. Sayed, Neveen A. Ashaat, Mohammed Gamal, Dalia Farouk Hussen, Soha Abuelela, Abeer Ramadan, Maha M. Kobesiy, Mai M. Shaker, Howida Elgebaly, Hala G. Elnady, Mona El Gammal, Engy A. Ashaat

**Affiliations:** 1 Medical Molecular Genetics Department, Human Genetics and Genome Research Institute, National Research Centre, Cairo, Egypt; 2 Community Medicine Research Department/Medical Research and Clinical Studies Institute, National Research Centre, Cairo, Egypt; 3 Department of Biochemistry and Molecular Biology, Faculty of Pharmacy (Girls), Al-Azhar University, Cairo, Egypt; 4 Professor of Human Genetics, and Biotechnology, Ain Shams University, Cairo, Egypt; 5 Child Health Department, Medical Research and Clinical Studies Institute, National Research Centre, Cairo, Egypt; 6 Human Cytogenetic Department, Human Genetics and Genome Research Institute, National Research Centre, Cairo, Egypt; 7 Clinical Pathology Department, Faculty of Medicine, Ain Shams University, Cairo, Egypt; 8 Molecular Genetics and Enzymology Department, Human Genetics and Genome Research Institute, National Research Centre, Cairo, Egypt; 9 Prenatal Diagnosis and Fetal Medicine Department, Human Genetics and Genome Research Institute, National Research Centre, Cairo, Egypt; 10 Professor of Pediatrics, Dean of Faculty of Post Graduate Childhood Studies, Ain Shams University, Cairo, Egypt; 11 Clinical Genetics Department, Human Genetics and Genome Research Institute, National Research Centre, Cairo, Egypt

**Keywords:** Inherited kidney diseases, NGS, Genetic variants, Consanguinity, Egyptian population

## Abstract

**Background:**

Inherited kidney diseases (IKDs) are a significant cause of chronic kidney disease (CKD) and end-stage kidney disease (ESKD), especially in children. While next-generation sequencing (NGS) has enhanced IKD diagnosis, data from consanguineous populations, where autosomal recessive inheritance is more common, remain limited.

**Aim:**

This study aimed to identify genetic variants associated with IKDs, primarily from consanguineous Egyptian families, using targeted next-generation sequencing (NGS). It further assessed genotype–phenotype correlations and explored clinical implications for early diagnosis, familial screening, and disease management.

**Methods:**

Twenty-six Egyptian patients clinically suspicion with IKDs were enrolled. Targeted NGS was conducted using a gene panel associated with IKDs. Variants were classified per American College of Medical Genetics and Genomics (ACMG) guidelines. Segregation analysis was performed when possible. In silico tools, including VarSome, I-Mutant 2.0, and GeneMANIA, were used to predict variant pathogenicity, protein impact, and gene–gene interactions.

**Results:**

Seventeen distinct variants were detected in 12 genes, including six novel mutations. Alport Syndrome was the most frequent disorder, with *COL4A3* and *COL4A5* mutations predominating. A novel *COL4A3* variant (c.3926C > A) was identified, reinforcing the role of collagen gene mutations. *FREM1* variants, including two novel ones, were linked to syndromic IKDs. *AGT* and *ACE* variants were associated with renal tubular dysgenesis, while *PKD1* and *PKHD1* mutations indicated both dominant and recessive polycystic kidney disease. High consanguinity supported autosomal recessive patterns.

**Conclusions:**

This study expands the mutational spectrum of IKDs in an underrepresented population and highlights the utility of targeted NGS in guiding early diagnosis, genetic counseling, and personalized management in high-risk, consanguineous populations.

**Supplementary Information:**

The online version contains supplementary material available at 10.1186/s40348-025-00203-2.

## Introduction

Chronic kidney disease (CKD) is a global health burden affecting both children and adults, ranking as the tenth leading cause of death worldwide in 2020, according to the World Health Organization (WHO) [1]. Projections suggest that by 2040, CKD will become the fifth leading cause of death [2]. In Egypt, CKD has seen a 36% increase in prevalence since 2009, becoming the fifth leading cause of death by 2019 [3], with costly hemodialysis remaining the primary treatment modality under national guidelines [4].

Chronic kidney disease (CKD) is commonly diagnosed using established clinical biomarkers. The most accurate indicator of kidney function is the glomerular filtration rate (GFR); however, due to the impracticality of direct measurement, GFR is typically estimated (eGFR) using equations based on endogenous markers such as serum creatinine (SCr) and cystatin C (CysC). Serum creatinine itself is directly measured in the laboratory, while eGFR provides a calculated estimate of renal function*.* Other indicators, including albuminuria, have demonstrated strong prognostic value and may precede a measurable decline in kidney function [5, 6]. However, these biomarkers have limited utility in determining the underlying etiology of CKD and its potential progression to end-stage kidney disease (ESKD) [7–9]. Among the key contributors to CKD, inherited kidney diseases (IKDs) account for a significant proportion, affecting up to 10% of adults and more than 70% of children who develop ESKD [10]. IKDs encompass a range of genetically heterogeneous disorders, exhibiting variability in age of onset, severity, and progression [11, 12]. Among congenital and childhood-onset renal conditions, inherited forms of nephrotic syndrome, particularly steroid-resistant subtypes or those with FSGS histology, are increasingly identified as monogenic IKDs [13–15].

To date, more than 150 IKDs have been classified into two primary categories**:** inherited kidney dysfunction and structural abnormalities [16, 17]. Over 400 genes have been implicated in these conditions, with disease-causing variants first reported in Alport Syndrome (AS) in 1990 [18, 19]. While some kidney disorders can be attributed to single-gene mutations, many follow complex inheritance patterns, suggesting multigenic etiologies. Traditional genetic screening methods, such as Sanger sequencing, have been limited in scope, typically focusing on a single gene at a time. In contrast, Next-Generation Sequencing (NGS) has significantly improved diagnostic yield in genetically heterogeneous diseases, enabling the simultaneous analysis of multiple kidney-related genes in a single experiment [20].

This study aimed to investigate the genetic etiology of IKDs in a cohort of Egyptian patients using targeted NGS. By employing comprehensive genetic screening, we sought to identify pathogenic and novel variants contributing to IKDs, assess genotype–phenotype correlations, and explore their clinical implications for early diagnosis and familial risk assessment.

## Patients and methods

### Ethical approval

This study was conducted in accordance with the ethical guidelines of the Declaration of Helsinki (1964) and was also approved by the Medical Ethics Committee of the National Research Centre (NRC), Cairo, Egypt (Number: 14447082021). Before entering the study, informed consents were obtained from all participants, for performing medical examinations and genetic screening. Efforts were made to ensure full understanding of study procedures in accordance with culturally appropriate consent practices in Egypt [21].

### Patients’ recruitment

Sixteen unrelated Egyptian families were referred to the Clinical Genetics Clinic at the National Research Centre (NRC), Egypt between December 2022 and December 2023, including 26 probands clinically diagnosed with inherited kidney diseases (IKDs). All patients were evaluated based on standardized diagnostic criteria, including clinical examination, laboratory tests, and imaging when necessary (e.g., renal ultrasound, biopsy for specific cases) to confirm structural or functional kidney abnormalities. We acknowledge the age variability among patients in this study, which ranged from neonates to 47 years. Accordingly, the cohort included both pediatric and adult cases. This heterogeneity was carefully considered during the clinical evaluation, variant interpretation, and genotype–phenotype correlation analysis. To exclude acquired causes of kidney disease, patients with probable immune disorders, diabetes, inflammatory processes, infectious diseases, or drug-induced nephropathy were excluded from the study.

### Clinical and family history assessment

Each patient underwent a comprehensive medical history evaluation, including age, sex, demographic data, age at onset, clinical symptoms, laboratory findings, and response to previous treatments. A detailed family history assessment was conducted, including the presence of similar illnesses in relatives, history of consanguinity among parents, and a three-generation pedigree analysis to assess inheritance patterns. Consanguinity was defined as a union between individuals related as second cousins or closer, with first-cousin marriages being most common in high-consanguinity populations like Egypt [22, 23].

Furthermore, secondary impairment of fetal kidney development due to placental insufficiency was excluded in the mothers through thrombophilia testing for common thrombophilia-associated genes.

### Blood samples

Peripheral blood samples (3 mL) were collected from each patient and available family members (parents, affected, and unaffected siblings) for genetic analysis. Blood samples were stored at −80 °C or immediately processed for DNA extraction to ensure optimal DNA integrity.

### Cytogenetic analyses

G-banding karyotyping was performed for all probands to assess potential chromosomal abnormalities that may contribute to inherited kidney disease phenotypes. This step was included to exclude structural or numerical chromosomal abnormalities in cases where syndromic features were suspected (e.g., microdeletions or aneuploidy affecting kidney development) and to differentiate monogenic kidney disorders from complex genetic conditions [24]. Approximately 30 metaphases were analyzed per patient, following the guidelines of the International System for Human Cytogenetic Nomenclature (ISCN 2020) [25]. Only patients with a normal karyotype proceeded to molecular genetic testing.

### Molecular genetic studies

#### Next-Generation Sequencing (NGS) and data analysis

Peripheral blood samples (~ 3 mL) were collected in EDTA tubes, and genomic DNA was extracted using the QIAamp DNA Blood Kit (Qiagen, Hilden, Germany) following the manufacturer's protocol.

A custom-targeted gene panel was designed to cover exonic and adjacent intronic regions of genes known to be associated with inherited kidney diseases. The selected genes were curated based on previous reports of monogenic kidney disorders and included in-house data on Egyptian patients. Exome library preparation was performed using the SureSelect Human All Exon V7 kit (Agilent Technologies, Santa Clara, California, USA), and sequencing was performed on the Illumina NovaSeq 6000 platform (Illumina, San Diego, CA, USA) to achieve an average coverage depth of ≥ 100x, ensuring high sequencing quality.

Raw sequencing reads were processed using the Genome Analysis Toolkit (GATK), following best-practice workflows for variant calling [26]. The sequencing data were converted from BCL to FASTQ files using the Bcl2fastq software, and reads were aligned to the human reference genome (hg19/GRCh37) using Burrows-Wheeler Aligner (BWA). Variant calling was performed using GATK Haplotype Caller, and filtering criteria included:

- Variants with MAF > 1% in global or regional databases (gnomAD, ExAC, 1000G, internal Egyptian dataset) were excluded. However, exceptions were made for variants known to be pathogenic or likely pathogenic in specific clinical or population contexts [27].
- Inclusion of variants causing missense, nonsense, frameshift, or splicing changes.
- Filtering out synonymous variants unless affecting splicing regulatory regions.
- Evaluation of pathogenicity following American College of Medical Genetics (ACMG) classification criteria [28].

Variant annotation was performed using ANNOVAR, integrating multiple databases for functional annotation, including dbSNP, ClinVar, and in-house databases.

## Validation and segregation analysis by Sanger sequencing

To confirm the pathogenicity of candidate variants, Sanger sequencing was performed on probands and their available family members for segregation analysis. Primer sequences flanking the identified variants were designed using Primer3 software, ensuring optimal primer specificity and amplification efficiency. PCR amplification was carried out using standard thermal cycling conditions, and PCR products were purified before sequencing with the BigDye Terminator v3.1 Cycle Sequencing Kit (Applied Biosystems, Foster City, CA, USA). Sequencing was conducted on the ABI Prism 3500 Genetic Analyzer (Applied Biosystems), and variant sequences were aligned to the reference genome using the BLASTN program for mutation confirmation.

### Functional prediction of identified variants

#### Computational prediction of deleterious variants

To assess the potential pathogenicity of the identified variants, in silico tools were used to predict their functional effects on protein structure and function. VarSome (https://varsome.com) was used for comprehensive variant annotation, integrating multiple prediction models (e.g., SIFT, PolyPhen-2) and databases to assess conservation, pathogenicity scores, and gene-disease associations [29, 30]. Additionally, SpliceAI was used to evaluate the potential effects of variants on splicing, allowing for the identification of possible splice-altering mutations.

#### Protein stability prediction

DynaMut2 (https://biosig.lab.uq.edu.au/dynamut2/), mCSM (https://biosig.lab.uq.edu.au/mcsm/stability), and I-Mutant 2.0 (https://folding.biofold.org/i-mutant/i-mutant2.0.html) were employed to predict the impact of mutations on protein stability. These tools estimate changes in Gibbs free energy (ΔΔG) by measuring the difference in energy between the folded and unfolded states. It should be noted that each predicted tool has a definite score and threshold to evaluate the mutation to be either stabilizing or destabilizing to the protein structure. To support structural analysis, we obtained the predicted protein structure from the AlphaFold Protein Structure Database using the corresponding UniProt ID, and the downloaded PDB file was used as input for DynaMut2 and mCSM analyses.

Predictions were classified as:

- Largely stable (ΔΔG > 0.5 kcal/mol)
- Largely unstable (ΔΔG < − 0.5 kcal/mol)
- Neutral (−0.5 ≤ ΔΔG ≤ 0.5 kcal/mol)

#### Gene–gene interaction network

To explore the biological relevance of identified variants, protein–protein interaction (PPI) networks were analyzed using GeneMANIA (http://www.genemania.org) [31]. The network identified co-expression, physical interactions, and shared pathways among key genes (e.g., *COL4A3, COL4A5, FREM1*), supporting their collective role in renal development and basement membrane biology.

## Results

### Patients’ characteristics

The Egyptian cohort comprised 26 patients from 16 unrelated families, clinically diagnosed with inherited kidney diseases (IKDs). The cohort included 15 males (57.69%) and 11 females (42.31%). with ages ranging from 1 week to 47 years and a median age of 13 years. Due to the descriptive and exploratory nature of the study and limited cohort size, no formal statistical testing was conducted; instead, narrative summaries and descriptive statistics were used to characterize clinical and genetic profiles and explore genotype–phenotype correlations.

**Fig. 1:**
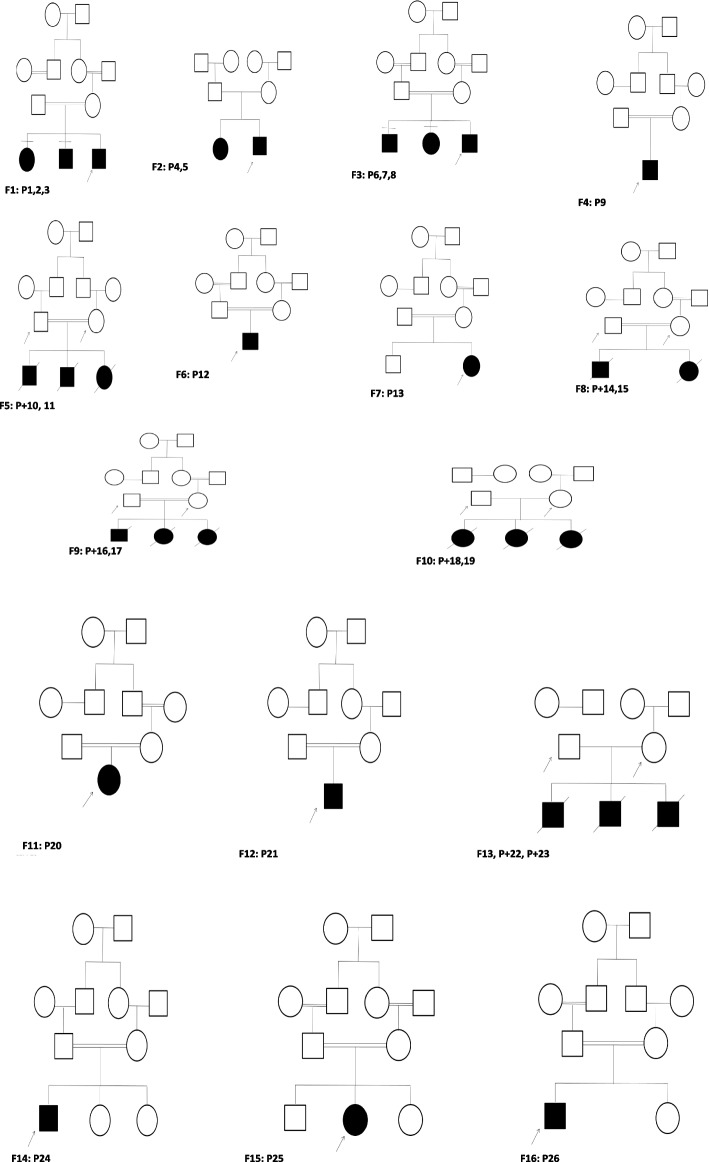
(I-II) Pedigree analysis figure for the 16 families reported in your study

Consanguinity was documented in 76.2% of the families, with confirmed first-degree parental consanguinity in most of these cases. A detailed clinical description of all patients is presented in Table 1, and pedigree structures illustrating affected and unaffected individuals across the 16 families are shown in Fig. 1 (I-II).

**Table 1:** Clinical and Genetic Characteristics of Study Subjects

Patient ID + *	Sex	Age	Consanguinity/Affected Family	Clinical Features	Renal Disorder	Eye Disorder	Fetal US	Genetic Variant (Gene/cDNA/Protein/Zygosity)
**Family 1**								
P1	F	16y	+ ve 1 st deg/+ ve	SNHL, hematuria, hypertension	End-stage renal failure	cataract, myopia	NA	COL4A5/c.4706G > A/p.Arg1569Gln/Hemizygous
P2	M	9y		SNHL		NA		
P3	M	13y		SNHL, hypertension		Myopia		
**Family 2**								
P4	M	3y	-ve/+ ve	SNHL, hematuria, visual defect	Focal segmental glomerulosclerosis	Retinitis pigmentosa	NA	COL4A3/c.2371C > T/p.Arg791Ter/Heterozygous
P5	F	6y	-ve/+ ve	SNHL	Renal failure	Myopia		
**Family 3**								
P6	M	4y	+ ve 1 st deg/+ ve	Deafness, renal disorder	Renal failure	NA	NA	COL4A3/c.3829G > A/p.Gly1277Ser/Heterozygous
P7	F	6y				Myopia		
P8	M	9y			End-stage renal failure	Squint		
**Family 4**								
P9	M	8y	+ ve 1 st deg/-ve	Renal disorder	End-stage renal failure	NA	NA	COL4A3/c.3926C > A/p.Pro1309His/Heterozygous
**Family 5**								
P10 +	F	36y	+ ve 1 st deg/+ ve	History of IUFDs with renal anomalies	Renal anomalies (small kidney)	NA	Oligo-hydramnios, IUFR, absent right kidney, left hypoplastic kidney	FREM1/c.4346G > A/p.Gly1449Glu/Heterozygous
P11 +	M	40y						
**Family 6**								
P12	M	1 m	+ ve 1 st deg/-ve	MCAs (renal anomalies, bifid nose, skeletal)	Left renal agenesis	NA	NA	FREM1/c.4382 T > G/p.Val1461Gly/Homozygous
**Family 7**								
P13	F	1w	+ ve 1 st deg/-ve	MCAs, bifid nose, FTT, renal anomalies	Absent left kidney	NA	Oligo-hydramnios, IUGR	FREM1/c.6373G > T/p.Glu2125Ter/Homozygous
**Family 8**								
P14 +	F	28y	+ ve 1 st deg/+ ve	History of IUFDs with renal anomalies	Renal tubular dysgenesis	NA	Oligo-hydramnios, renal tubular dysgenesis	ACE/c.38_49Del/p.Leu13_Leu16Del/Heterozygous
P15 +	M	33y						
**Family 9**								
P16 +	F	30y	+ ve 1 st deg/+ ve	History of IUFDs with renal anomalies	Renal tubular dysgenesis	NA	renal tubular dysgenesis	AGT/c.110A > G/p.Tyr37Cys/Heterozygous
P17 +	M	38y						
**Family 10**								
P18 +	F	32y	-ve/+ ve	Polycystic kidney disease	Polycystic kidney	NA	NA	PKD1/c.9013C > T/p.Gln3005Ter/Heterozygous
P19 +	M	42y						
**Family 11**								
P20	F	9y	+ ve 1 st deg/-ve	Renal disorder, hepatomegaly	Polycystic kidney	NA	NA	PKHD1/c.4870C > T/p.Arg1624Trp/Homozygous
**Family 12**								
P21	M	10y	+ ve 1 st deg/-ve	Renal failure	End-stage renal failure	NA	NA	NPHS2/c.163G > A, c.275-44G > C/Compound Heterozygous
**Family 13**								
P22 +	F	36y	-ve/+ ve	History of IUFDs with renal anomalies	Right renal agenesis	NA	IUGR, oligo-hydramnios	TBC1D8B/c.827 + 12 T > C/Hemizygous
P23 +	M	47y						
**Family 14**								
P24	M	7y	+ ve 1 st deg/-ve	Renal disorder	Right dysplastic kidney	NA	NA	NOTCH2/c.2503G > T/p.Ala835Ser/Heterozygous
**Family 15**								
P25	F	6y	+ ve 1 st deg/-ve	MCAs (renal anomalies, obesity, CHD, polydactyly)	Bilateral renal cysts	Retinitis pigmentosa	NA	BBS4/c.172C > T/p.Gln58Ter/Homozygous
**Family 16**								
P26	M	1y	+ ve 1 st deg/-ve	FTT, renal disorder	Nephro-calcinosis	NA	IUGR, oligo-hydramnios	CYP24A1/c.428_430del/p.Glu143del/Homozygous

P+ Individuals with history of IUFD evaluated for carrier status, not clinically assessed patients. *All Patient IDs (P1–P26, P +) correspond directly to the labels in Fig. 1, ensuring cross-reference between the table and pedigree structures

*Abbreviations*: *P* Patient, *P +* Parent, *M* male, *F* female, *y* year(s), *w* week(s), *SNHL* Sensorineural hearing loss, *IUGR* Intrauterine Growth Restriction, *CHD* Coronary Heart Disease, *FTT* Failure to thrive, + ve Positive, -ve Negative, *NA* Not available, *MCA* multiple congenital anomalies, *ESRF* End-stage renal failure

### Renal disorders as a dominant feature

All patients presented with renal abnormalities, including structural anomalies (e.g., agenesis, dysplasia, cysts), tubular defects, and progressive renal impairment. Multiple patients (e.g., P1, P5, P6, P8, P9, P21) had progressed to end-stage renal failure (ESRF). Polycystic kidney disease (PKD) was diagnosed in P15, P18, and P20, each carrying variants in PKD1, *PKHD*1, or with syndromic features suggestive of cystic pathologies.

### Syndromic and multisystem involvement

A significant subset of patients exhibited syndromic features. Sensorineural hearing loss (SNHL) was frequent in families 1–3 (P1–P5), aligning with diagnoses of Alport Syndrome. Visual defects (e.g., cataract, myopia, retinitis pigmentosa) were noted in P4, P5, and P20. Multisystem involvement was evident in patients with *FREM1* or *BBS4* variants (e.g., P12, P13, P25), presenting with bifid nose, skeletal anomalies, obesity, polydactyly, or congenital heart disease (CHD). Hepatomegaly and failure to thrive were observed in P20 and P26, respectively.

### Prenatal and perinatal findings

Five patients (P10, P11, P13, P14, P22) had prenatal histories notable for intrauterine fetal demise (IUFD), oligohydramnios, or structural anomalies on fetal ultrasound. These included renal agenesis, tubular dysgenesis, and absent or dysplastic kidneys. These findings underscore the importance of considering inherited kidney disease in fetal presentations with renal anomalies.

### Genetic and familial patterns

The pedigree diagrams Fig. 1 (I-II) support the likelihood of autosomal recessive inheritance in a large proportion of cases. Specifically, multiple affected siblings were observed in several families, notably in Families 1 (P1–P3), 2 (P4–P5), 3 (P6–P8), and 5 (P10–P11), with unaffected or heterozygous parents, consistent with recessive disease segregation. This observation is further reinforced by the high rate of consanguinity (76.2%), with 73% of consanguineous families reporting first-cousin relationships.

Additionally, the pedigree structures reveal vertical transmission in select families, suggestive of autosomal dominant inheritance patterns. In Family 10 (P18–P19) and Family 11 (P20), the affected individuals carry heterozygous variants in genes (*PKD1* and *PKHD1*, respectively) commonly associated with dominant and recessive polycystic kidney disease phenotypes. Moreover, hemizygous X-linked patterns were noted in male patients such as P1 and P12–P13, supporting X-linked transmission, particularly in *COL4A5* and *FREM1*-related pathologies. Cytogenetic analysis confirmed normal karyotypes in all patients (46, XX or 46, XY), ruling out chromosomal abnormalities as contributors to the observed renal phenotypes.

### Molecular genetic findings

NGS identified 17 distinct variants across 12 genes with autosomal dominant (41.2%), autosomal recessive (41.2%), and X-linked (17.6%) inheritance. Variants were classified as pathogenic (52.9%), VUS (41.2%), or likely pathogenic (5.9%). Benign variants were initially detected but were subsequently filtered out during the GATK pipeline analysis, consistent with the methodology described.

Three patients from Family 1 (P1–P3) harbored a hemizygous *COL4A5* pathogenic variant (c.4706G > A), consistent with *X-*linked Alport syndrome. Patients from Family 2 (P4, P5) and Family 3 (P6–P8) carried *COL4A3* variants classified as either pathogenic or VUS, supporting collagen IV-related nephropathies. A compound heterozygous variant in *NPHS2* (c.163G > A, p. Gly55Arg and c.275-44G > C) was identified in P22, indicating steroid-resistant nephrotic syndrome*.* For Family 4 (P9), the *COL4A3* variant c.3926C > A (p. Pro1309His), classified as VUS, was confirmed to segregate with the phenotype: both parents were identified as heterozygous carriers, consistent with autosomal recessive inheritance. However, this segregation alone was insufficient to reclassify the variant without additional functional evidence.

Among the affected genes, *COL4A5* (X-linked) was found in one patient (5.9%), and *COL4A3* (autosomal dominant) in three patients (17.6%). The most recurrent mutation was *COL4A3* (c.2371C > T, p. Arg791Ter), a nonsense variant classified as pathogenic. *FREM1* mutations were observed in three unrelated cases (17.6%) presenting with bifid nose, anorectal, and renal anomalies. These included one likely pathogenic variant (c.6373G > T, p. Glu2125Ter) and two classified as VUS and pathogenic, respectively.

All patients clinically diagnosed with Alport syndrome also exhibited extrarenal manifestations, including sensorineural hearing loss, consistent with syndromic features of AS and supporting the pathogenic relevance of identified *COL4A3* and *COL4A5* variants.

Variants in *ACE* and *AGT*, associated with renal tubular dysgenesis, were classified as VUS (11.8%). Mutations in *PKD1* and *PKHD1* were identified in separate patients, representing autosomal dominant and autosomal recessive polycystic kidney disease (ADPKD and ARPKD), respectively. The *PKD1* variant (c.9013C > T, p. Gln3005Ter) and *PKHD1* variant (c.4870C > T, p. Arg1624Trp) were both pathogenic. Importantly, the PKD1 c.9013C > T variant does not correspond to any PKD1 pseudogenes, all of which carry a G at this position in multiple sequence alignment, supporting its validity as a true PKD1 finding rather than a pseudogene artifact.

Additionally, an X-linked mutation in *TBC1D8B* (5.9%) (c.827 + 12 T > C) was detected. P22, a 36-year-old woman with a history of intrauterine fetal demise (IUFD) but no family history of similar symptoms, harbored this novel intronic variant, classified as VUS. Although the NGS output labeled the variant as hemizygous, this patient is genetically female (46, XX), and the finding most likely represents a heterozygous state. Sanger sequencing could not be performed due to limited DNA availability and inability to recontact the patient, which we acknowledge as a limitation. SpliceAI analysis yielded a low splice acceptor loss score (0.03), suggesting minimal disruption. The absence of living offspring further precluded segregation analysis.

In nephrotic syndrome-related genes, *NPHS2* mutations accounted for 11.8% of variants, with a compound heterozygous mutation identified, consisting of a missense variant (c.163G > A, p. Gly55Arg) and a splice variant (c.275-44G > C), both classified as VUS (11.8%). As illustrated in method section, variants with a minor allele frequency (MAF) > 1% were excluded unless previously confirmed to be pathogenic or likely pathogenic. For example, Rood and his colleagues reported that *NPHS2* p. Arg229Gln (MAF ~ 3%) was retained due to its established pathogenicity when found in trans with specific mutations in exons 7 or 8 [27].

Other relevant findings included a *NOTCH2* missense variant (c.2503G > T, p. Ala835Ser), classified as VUS and associated with Alagille syndrome, a homozygous nonsense variant in *BBS4* (c.172C > T, p. Gln58Ter), and a pathogenic deletion in *CYP24A1* (c.428_430del, p. Glu143del), linked to familial hypercalcemia.

**Fig. 2:**
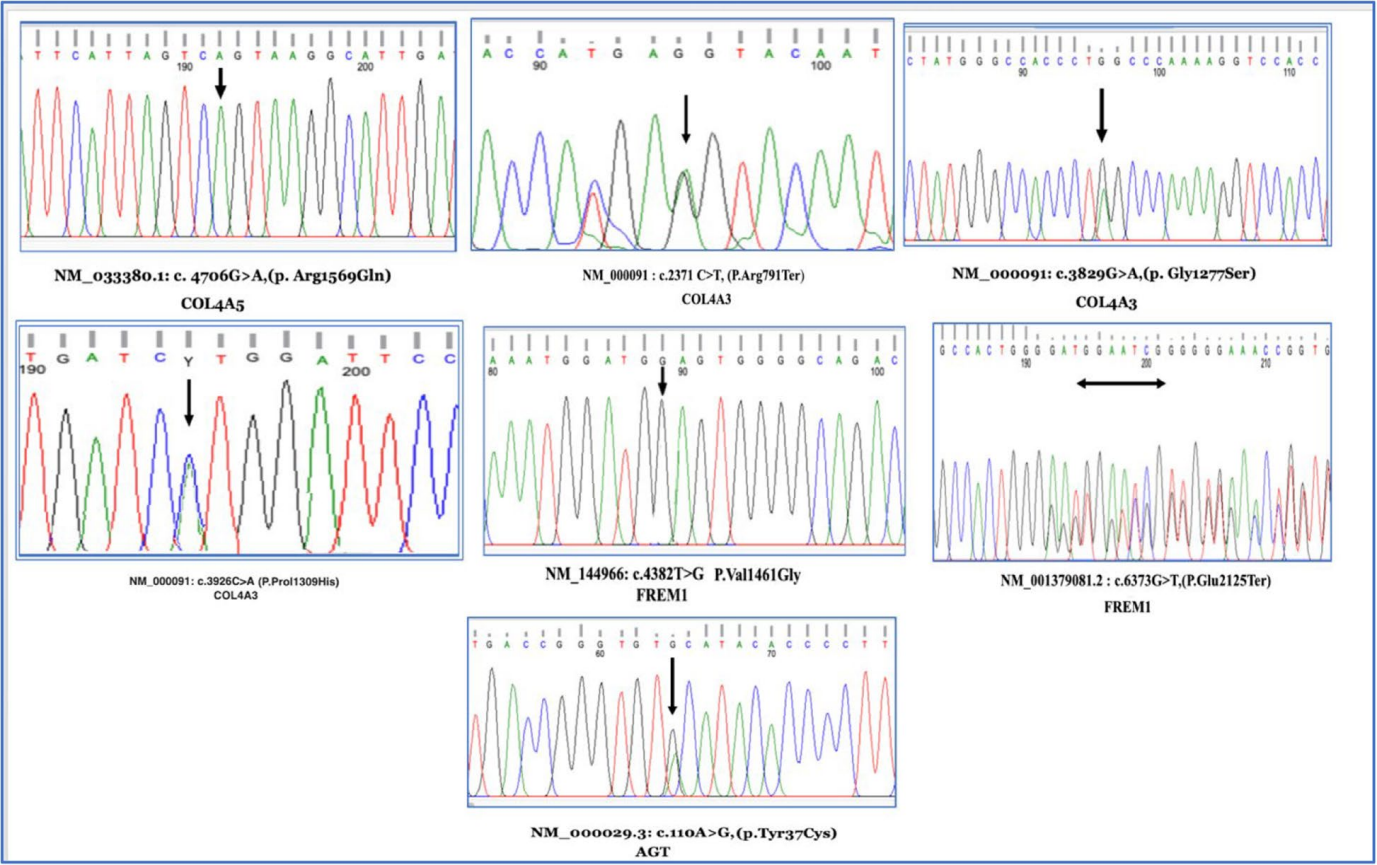
Sequencing chromatographs showing variants identified for patients who completed the follow-up (marked with the arrow)

Of the 17 total variants, six (41.2%) were novel, not reported in gnomAD, HGMD, or the 1000 Genomes Project, suggesting potential relevance to regional or founder effects in the Egyptian population. Table 2 provides details of these novel variants. Sanger sequencing confirmed variant segregation in families who completed clinical follow-up is presented in (Fig. 2).

**Table 2:** List of variants detected in our patients

Family ID	Patient ID	Affected Gene	Inheritance	OMIM Disease	Variant	Zygosity	Variant type	Sanger/Segregation analysis	VarSome/Classification ACMG	dbSNP ID	Ref
1	P1 P2 P3	*Col4A5*	XLD	Alport Syndrome	NM_033380.3: c.4706G > A, (p. Arg1569Gln)	Hemi	Missense	(mother + carrier)	Pathogenic PM1, PM2, PP3 + PP4 + PP1	rs281874743	[32]
2	P4 P5	*COL4A3*	AD	Alport syndrome	NM_000091.5: c.2371C > T, (p. Arg791Ter)	Hetero	Nonsense	(Probands + perants)	Pathogenic PVS1, PM2, PP5	rs1060499654	Reported in ClinVar [33–35]
3	P6 P7 P8	*COL4A3*	AD	Alport syndrome	NM_000091.5: c.3829G > A, (p. Gly1277Ser)	Hetero	Missense	(Probands + perants)	Pathogenic PM1, PM2, PP3, PP4	rs190598500	Reported in ClinVar [36]
4	P9	*COL4A3*	AD	Alport syndrome	NM_000091.5: c.3926C > A (p. Prol1309His)	Hetero	Missense	(Probands + parents) resolved	VUS PM2, PP3	**This study**	Not reported
5	P10, p11	*FREM1*	AR	Bifid nose with or without anorectal and renal anomalies	NM_144966.7: c.4346G > A, (p. Gly1449Glu)	Hetero	Missense	resolved	Pathogenic PM2, PP3, PP4	rs1850100613	Reported in dbSNP
6	P12	*FREM1*	AR	Bifid nose with or without anorectal and renal anomalies	NM_144966.7: c.4382 T > G p. Val1461Gly	Homo	Missense	(Proband) Partially resolved	VUS PM2, PP3	**This study**	Not reported
7	P13	*FREM1*	AR	Bifid nose with or without anorectal and renal anomalies	NM_144966.7: c.6373G > T (P. Glu2125Ter)	Homo	Nonsense	(Proband) Partially resolved	Likely Pathogenic PVS1, PM2, PP3	**This study**	Not reported
8	P14, P15	*ACE*	AR	Renal tubular dysgenesis	NM_000789.5: c.38 _49Del (p. Leu13_Leu16Del)	Hetero	Indel	Not resolved	VUS PM4, PM2	rs751352152	Reported in dbSNP
9	P16,p17	*AGT*	AR	Renal tubular dysgenesis	NM_000029.4: c.110A > G, (p. Tyr37Cys)	Hetero	Missense	Carrier Partially resolved	VUS PM2, PP3	**This study**	Not reported
10	P18, P19	*PKD1*	AD	Polycystic kidney disease 1	NM_001009944.2: c.9013C > T (p. Gln3005Ter)	Hetero	Nonsense	Not resolved	Pathogenic PVS1, PM2, PP5	**rs1063401**	Reported in clinvar
11	P20	*PKHD1*	AR	Autosomal recessive polycystic kidney disease	NM_138694.3: c.4870C > T (p. Arg1624Trp)	Homo	Missense	Not resolved	Pathogenic PM1, PM2, PP3, PP4	rs200391019	Reported in ClinVar [37–40]
12	P21	*NPHS2*	AR	Nephrotic syndrome, type 2	NM_014625.4: c.163G > A, (p.Gly55Arg) NM_014625.4: c.275-44G > C	Comp. Hetero	Missense Splice	Not resolved	VUS VUS PM2, PP3	rs1409794630 **This study**	Reported in dbSNP Not reported
13	P22, P23	*TBC1D8B*	XL	Nephrotic syndrome type 20	NM_017752.3: c.827 + 12 T > C p.?	Hemi	Splice	Not resolved	VUS BP4, PM2	**This study**	Not reported
14	P24	*NOTCH2*	AD	Alagille syndrome	NM_024408.3: c.2503G > T (p. Ala835Ser)	Hetero	Missense	Not resolved	VUS PM2, PP3	rs1553197411	Reported in dbSNP
15	P25	*BBS4*	AR	Ciliopathies/tubuloi nterstitial diseases Bardet-Biedl syndrome 4	NM_033028.5: c.172C > T (p. Gln58Ter)	Homo	Nonsense	Not resolved	Pathogenic PVS1, PM2, PP5	rs886039802	Reported in ClinVar [41]
16	P26	*CYP24A1*	AR	Familial hypercalcemia	NM_000782.5: c.428_430 del (p. Glu 143del)	Homo	Indel	Not resolved	Pathogenic PM4, PM2, PP3, PP4	rs777676129	Reported in ClinVar [42, 43]

*Abbreviations*: *AR* Autosomal Recessive, *AD* autosomal dominant, *XL* X-linked, *Homo.* Homozygous, *Hetero.* Heterozygous, *Hemi.* Hemizygous, *Comp. Hetero.* Compound Heterozygous, *VUS* variant of unknown significance, *ACMG* American College of Medical Genetics and Genomics, *COL4A5* Collagen type IV alpha 5 chain, *COL4A3* Collagen type IV alpha 3 chain, *FREM1* FRAS1-related extracellular matrix 1, *AGT* Angiotensinogen*, PKD1* Polycystin 1, *PKHD1* Polycystic kidney and hepatic disease 1, N*PHS2* NPHS2 stomatin family member, podocin, *TBC1D8B:* TBC1 domain family member 8B. *NOTCH2:* Notch receptor 2*, BBS4:* Bardet Biedl syndrome 4, *CYP24A1:* Cytochrome P450 family 24 subfamily A member 1

### Case resolution status

**Fig. 3:**
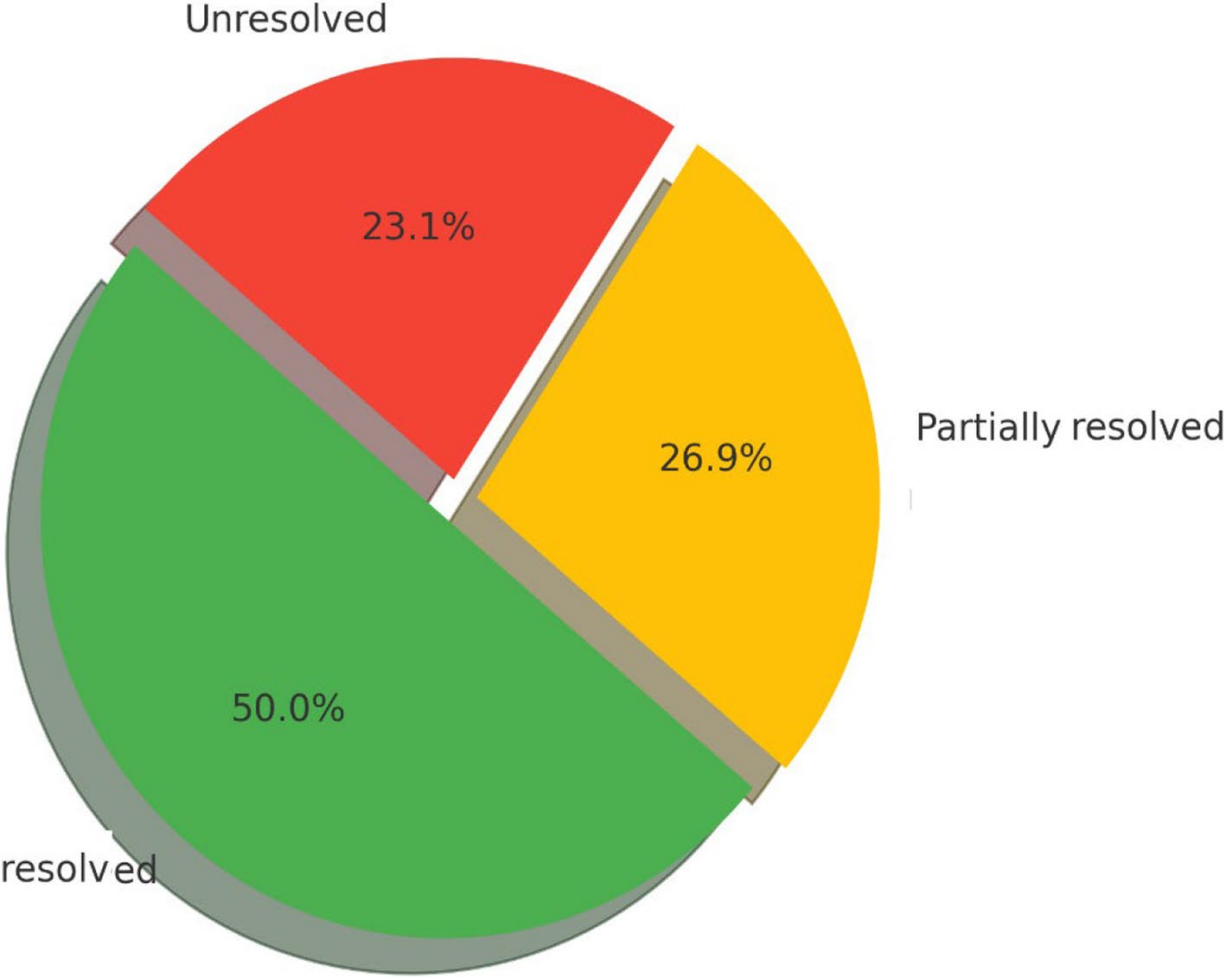
Distribution of genetic diagnosis outcomes in the study cohort

To assess the diagnostic yield, patients were classified based on variant pathogenicity, segregation analysis, and genotype–phenotype concordance. Of the 26 patients, 13 (50%) were considered genetically resolved, harboring pathogenic or likely pathogenic variants with consistent clinical correlation. Seven cases (26.9%) were deemed partially resolved, typically involving variants of uncertain significance (VUS) or single heterozygous variants in recessive genes. The remaining six patients (23.1%) were unresolved, with no definitive genotype–phenotype association identified. This categorization is detailed in Table 2 and Fig. 3.

### Prediction of deleterious variants

To enhance the interpretation of novel variants in the absence of functional assays, we employed multiple validated in silico tools to assess their potential pathogenicity. Among the six novel variants identified in our cohort, four were missense mutations and two were intronic/splice-site changes. These were specifically analyzed using VarSome for ACMG classification, SpliceAI for splicing impact, and I-Mutant 2.0, DynaMut2, and mCSM for protein stability predictions (Table 3)***.***

**Table 3:** In silico analysis of the identified missense variants in target genes

Gene	Variant	dbSNP ID	VarSome Classification	Prediction on Protein Stability			
				**SubstitutionWt/Mut**	**I-Mutant Stability**	**RI**	**DDG kcal/mol**
**Col4A5** **NM_033380.1**	c.4706G > A	rs281874743	Pathogenic	Arg **/** Gln	Decrease	8	−2.47
**COL4A3** **NM_000091**	c.3829G > A	rs190598500	Pathogenic	Gly**/**Ser	Decrease	4	0.05
	c.3926C > A	This study	VUS	Pro/His	Decrease	1	−0.67
***FREM1*** **NM_144966.7**	c.4346G > A	rs1850100613	Pathogenic	Gly/Glu	Decrease	9	−3.97
	c.4382 T > G	This study	VUS	Val/Gly	Decrease	9	−1.96
**AGT** **NM_000029.3**	c.110A > G	This study	VUS	Tyr/Cys	Decrease	6	−2.67
**PKHD1** **NM_138694.3**	c.4870C > T	rs200391019	Pathogenic	Arg/Trp	Decrease	8	−2.72
**NPHS2** **NM_014625.2**	c.163G > A	rs1409794630	VUS	Gly/Arg	Decrease	8	−1.22
**NOTCH2** **NM_024408.3**	c.2503G > T	rs1553197411	VUS	Ala/Ser	Decrease	3	0.26

*Abbreviations*: *VUS* Variant of Unknown Significance, *Wt/Mut* Wild/Mutant, *ΔΔG* Free energy change, *RI* Reliability Index (from 0–5 is unstable and from 6–9 is highly stable)

Most missense variants were classified as either pathogenic or variants of uncertain significance (VUS) based on VarSome annotations (Table 3). Confirmed pathogenic variants included *COL4A5* (c.4706G > A, p. Arg1569Gln), *COL4A3* (c.3829G > A, p. Gly1277Ser), *FREM1* (c.4346G > A, p. Gly1449Glu), and *PKHD1* (c.4870C > T, p. Arg1624Trp), reinforcing their established roles in syndromic and recessive kidney disorders.

Variants classified as VUS included *FREM1* (c.4382 T > G, p. Val1461Gly), *AGT* (c.110A > G, p. Tyr37Cys), *NPHS2* (c.163G > A, p. Gly55Arg), and *NOTCH2* (c.2503G > T, p. Ala835Ser). These included several of the novel variants and, despite the uncertain classification, showed consistent destabilizing effects on protein structure (negative ΔΔG) across all three prediction tools, indicating likely functional impairment (Table 3)*.* A novel *COL4A3* variant (c.3926C > A, p. Pro1309His) was also classified as VUS, highlighting the molecular heterogeneity of collagen IV-related nephropathies.

SpliceAI analysis of novel intronic variants revealed low scores for predicted splicing disruption. *NPHS2* (c.275-44G > C) and *TBC1D8B* (c.827 + 12 T > C), yielded scores of 0.01 (splice loss) and 0.03 (splice acceptor loss), respectively. Although these variants are novel, their low SpliceAI scores suggest a minimal likelihood of splice disruption.

### Prediction on protein stability

The potential impact of missense variants on protein thermodynamic stability was further evaluated by calculating changes in Gibbs free energy (ΔΔG) between wild-type and mutant forms. Across I-Mutant 2.0, mCSM, and DynaMut2, all missense variants exhibited negative ΔΔG values **(**Table 3**),** indicative of reduced protein stability. This structural destabilization supports the hypothesis that these variants may impair protein function and contribute to disease pathogenesis.

### Gene–gene interaction

To further contextualize the functional relevance of the identified variants, we explored gene–gene interaction network using GeneMANIA. Given that our NGS panel was curated to target genes implicated in inherited kidney diseases, it was expected that many of the genes would be functionally interconnected or participate in shared biological pathways. The resulting network revealed that *COL4A3, COL4A4, COL4A5,* and *FREM1* emerged as central nodes within a highly interconnected subnetwork. These genes were linked through multiple layers of association, including co-expression, physical interaction, and shared involvement in renal development, extracellular matrix assembly, and basement membrane stability **(**Supplementary Figure S1**)**.

This network analysis reinforces the functional significance of the identified variants by mapping their involvement in essential pathways related to glomerular filtration barrier integrity, nephrogenesis, and basement membrane maintenance. The central positioning of *COL4A* family genes and *FREM1* within the network further highlights their potential cooperative role in the pathogenesis of inherited kidney diseases (IKDs) in this cohort.

## Discussion

Inherited kidney diseases (IKDs) represent a major cause of chronic kidney disease (CKD) and progression to end-stage kidney disease (ESKD), particularly when onset occurs in childhood. While the global burden of IKDs is increasingly recognized, their characterization in high-consanguinity populations remains understudied. This study aimed to explore the genetic basis of IKDs among Egyptian patients recruited from consanguineous families with a clinical suspicion of inherited renal disorders. Given the targeted nature and limited sample size, our findings should be viewed as exploratory and hypothesis-generating rather than reflective of national incidence rates. Recent advances in molecular genetics have significantly enhanced our understanding of IKDs, allowing for earlier diagnosis and improved disease management. Among these advances, next-generation sequencing (NGS) has emerged as a powerful tool in identifying genetic causes of kidney disease at an early stage, which can facilitate timely interventions to delay or prevent ESKD progression [19, 44, 45]. Our study adds to this body of knowledge by applying targeted NGS and comprehensive bioinformatics analyses to a cohort of 26 Egyptian patients with clinically suspected IKDs, aiming to delineate pathogenic and novel variants across diverse clinical subtypes.

We identified 17 different variants; 9 pathogenic or likely pathogenic and 8 variants of uncertain significance (VUS) across 12 genes, including six novel variants. Benign variants were initially detected but were subsequently filtered out during the GATK pipeline, as described in the Methods, ensuring that only pathogenic, likely pathogenic, and VUS calls were retained for analysis. All retained variants were then evaluated using in silico tools to predict their pathogenicity, splicing potential, and protein destabilization, strengthening genotype–phenotype interpretations*.* The detailed clinical evaluation of our patients provided valuable genotype–phenotype correlations, particularly in Alport Syndrome (AS), syndromic kidney disorders, renal tubular dysgenesis, and polycystic kidney disease.

Analysis of family structures and clinical profiles revealed that 23 of 26 patients were from consanguineous unions, primarily first-degree cousin marriages (76.2%), highlighting the high background rate of consanguinity in the cohort. Families 1–5, 7, and 13 presented with more than one affected sibling born to unaffected parents, a pedigree pattern that strongly supports autosomal recessive inheritance, particularly in consanguineous unions where pathogenic variants in *COL4A5*, *AGT, PKHD1*, and *FREM1* were identified [13–46].

The carrier status observed in female patients with renal anomalies (F5, F8, F9, F10, F13) and the normal cytogenetic findings (46, XX and 46, XY) further support a monogenic etiology and exclude chromosomal causes in syndromic presentations. These inheritance patterns align with prior studies showing increased homozygosity and AR disease burden in high-consanguinity populations such as Egypt and other MENA countries [47, 48].

### Alport syndrome and collagen mutations

Our study identified three known genetic variants and one novel variant in *COL4A3* (c.2371 C > T, c.3829G > A, and c.3926C > A, respectively), in addition to a *COL4A5* variant (c.4706G > A), which was associated with AS the most prevalent disorder in our cohort. The novel *COL4A3* variant (c.3926C > A) is particularly noteworthy, as a similar alteration (c.3926C > T, p. Pro1309Leu) has been previously reported as pathogenic for AS. Computational predictions further supported its pathogenicity through protein destabilization (negative ΔΔG) and ACMG classification as a VUS requiring further functional validation. Although segregation analysis in Family 4 confirmed that the *COL4A3* VUS (p. Pro1309His) was inherited in an autosomal recessive manner, this alone was insufficient to reclassify the variant without further functional or population-based evidence, highlighting the challenges in interpreting VUS even when familial patterns are consistent. These findings reinforce the clinical importance of *COL4A3* and *COL4A5* mutations in Egyptian patients with IKDs, particularly AS.

AS is a progressive hereditary kidney disorder caused by pathogenic alterations in *COL4A3*, *COL4A4*, and *COL4A5*, which encode the type IV collagen (α3, α4, and α5) chains, respectively [44, 49]. The disease is considered the second most prevalent IKD after autosomal dominant polycystic kidney disease (ADPKD) in Western populations [42, 50]. Approximately 80% of AS cases are X-linked (XLAS), most commonly due to *COL4A5* mutations [45], leading to heterogeneous renal manifestations. In males with XLAS, genotype–phenotype correlations indicate that missense mutations are associated with mild phenotypes, splicing variants with moderate phenotypes, and nonsense mutations with the most severe renal dysfunction [51, 52]. Given these genotype–phenotype correlations, our study emphasizes the diagnostic value of sequencing especially in consanguineous populations with high recurrence rates.

### FREM1 variants and syndromic kidney disorders

Our study identified three missense variants in *FREM1* (c.4346G > A, c.4382 T > G, c.6373G > T), two of which were novel. Alterations in *FREM1* have been linked to Bifid Nose, Renal Agenesis, and Anorectal Malformations (BNAR) syndrome, a rare autosomal recessive disorder [53]. Notably, *FREM1* and *FREM2* mutations have also been associated with congenital anomalies of the kidney and urinary tract (CAKUT) [54, 55], highlighting the crucial role of *FREM* genes in renal organogenesis [56]. The novel FREM1 variants showed destabilizing ΔΔG values and were classified as either VUS or likely pathogenic, suggesting a potential disruptive effect on protein structure and function. The identification of two novel *FREM1* variants suggests a previously unreported genetic contribution to syndromic renal diseases in our cohort.

### Renal tubular dysgenesis and RAAS-related variants

In addition to AS and syndromic kidney disorders, we identified two genetic variants associated with renal tubular dysgenesis, a severe disorder characterized by anuria, hypotension, and abnormal kidney development in utero. These included a novel missense variant (c.110A > G, p. Tyr37Cys) in *AGT* and an indel variant (c.38_49Del, p. Leu13_Leu16Del) in *ACE*. Given the critical role of the renin–angiotensin–aldosterone system (RAAS) in blood pressure regulation and kidney function, variants in *AGT* and *ACE* are strong candidates for modifying renal outcomes in affected individuals.

RAAS is an essential system for maintaining blood pressure, renal hemodynamics, and electrolyte balance [57]. Dysregulation of this system leads to pathological changes in kidney function, contributing to renal failure progression. Studies have shown that genetic variants in RAAS components, including *AGT*, can influence disease severity and outcomes [58, 59]. Notably, in 2017, Nuglozeh Edem et al. identified six *AGT* variants associated with renal tubular dysgenesis, including a single nucleotide polymorphism (SNP, rs699) in exon 2 that increases angiotensinogen production [60]. Our findings of a novel AGT variant classified as a VUS with destabilizing effects support its candidacy as a pathogenic modifier and warrant further functional studies.

### Polycystic kidney disease and other monogenic IKDs

We identified two disease-causing variants in *PKD1* (c.9013C > T, p. Gln3005Ter) and *PKHD1* (c.4870C > T, p. Arg1624Trp), associated with *ADPKD* and autosomal recessive polycystic kidney disease (ARPKD), respectively. In silico analysis using VarSome classified both variants as pathogenic. Notably, the *PKD1* variant (c.9013C > T, p. Gln3005Ter) was absent in public databases such as ExAC, dbSNP, and gnomAD, reinforcing its potential significance as a novel pathogenic mutation. Due to PKD1's genomic complexity and the presence of pseudogenes, accurate detection of disease-causing mutations remains challenging [61]. The use of targeted NGS combined with strict variant filtering and orthogonal confirmation (Fig. 2) enhances diagnostic precision in complex genomic regions like *PKD1*.

We appreciate the challenges associated with interpreting *PKD1* variants due to the presence of highly homologous pseudogenes. In the case of P15, the novel *PKD1* variant was identified using a high-confidence NGS panel designed to minimize misalignment with pseudogenes. However, due to limited DNA availability and resource constraints, Sanger sequencing and segregation analysis were not performed.

### Clinical implications and future directions

The use of comprehensive genetic panels for diagnosing inherited kidney diseases (IKDs) offers significant advantages, particularly in cases with overlapping clinical features [62, 63]. For example, a recent study utilizing a 177 gene panel targeting glomerulopathies, CAKUT, and ciliopathies/tubulointerstitial diseases achieved a 43% diagnostic yield [64]. Consistent with these findings, our study demonstrates that phenotype-driven targeted NGS panels are particularly valuable in high-consanguinity populations, such as Egypt, where autosomal recessive diseases are more prevalent. This supports previous evidence highlighting the efficiency of targeted screening approaches in similar settings [65].

Identifying monogenic causes of kidney disease enables early risk assessment for asymptomatic family members through extended family screening. This facilitates timely interventions, supports informed reproductive decisions, and provides a foundation for genetic counseling. The high rate of consanguinity observed in this cohort likely contributes to the increased detection of autosomal recessive (AR) inherited kidney disorders, as consanguineous unions elevate the probability of homozygosity for rare deleterious variants inherited from a common ancestor [66, 67]. However, while consanguinity increases the genetic risk for AR conditions, it does not independently determine disease incidence, which is also influenced by variant frequency, penetrance, and other modifying factors [48]. These findings justify the need for community-based carrier screening and premarital counseling strategies aimed at reducing the incidence of inherited renal disorders in high-risk groups.

In line with ACMG/AMP recommendations, classification of a case as genetically resolved requires robust supporting evidence, including pathogenicity classification, phenotypic correlation, and, ideally, segregation or functional validation. In cases such as P22, where the NGS pipeline reported a hemizygous call in a genetically female patient, the most likely explanation is a heterozygous state. However, without confirmatory Sanger sequencing, such findings must be interpreted with caution. This highlights the need for orthogonal validation in future studies to confirm zygosity and strengthen variant classification. Although the variant detected in P21 (rs777676129) is labeled as pathogenic in ClinVar and exhibits low allele frequency, the absence of segregation data and functional confirmation led us to conservatively classify this case as unresolved. This highlights the importance of integrating multiple lines of evidence when interpreting genetic findings in clinical research settings [28].

The discovery of six novel variants in our cohort emphasizes the need for functional characterization and validation to confirm their pathogenicity. These findings also highlight the current underrepresentation of Middle Eastern and North African populations in global genetic databases. Expanding region-specific genetic data will be essential to improve diagnostic interpretation, refine genotype–phenotype correlations, and enhance personalized therapeutic strategies [68]. Such population-level efforts are critical in aligning with recent global initiatives that seek to map rare variants influencing chronic kidney disease (CKD) susceptibility and progression.

Moreover, early genetic diagnosis not only allows for timely therapeutic intervention and progression monitoring but also aids in the identification of asymptomatic or mildly affected carriers. This opens new avenues for prenatal diagnosis, preimplantation genetic testing, and other preventive measures, ultimately contributing to better long-term outcomes.

Incorporating genetic testing into routine nephrology practice especially in pediatric and consanguineous populations has the potential to transform clinical management of IKDs by enabling precision diagnostics, risk-based surveillance, and family-centered care.

### Study strengths

This study provides valuable genetic insights into IKDs in an Egyptian cohort, leveraging targeted NGS to identify 17 distinct variants across 12 genes, including six novel variants. A key strength of this study is its comprehensive genetic screening approach, which enabled the detection of rare and novel disease-causing mutations, thereby contributing to the expansion of the mutational spectrum of IKDs in an underrepresented population [69], where exome and panel-based sequencing are increasingly reshaping nephrology diagnostics. The well-characterized patient cohort, consisting of clinically diagnosed cases with detailed phenotype-genotype correlations, further strengthens the study’s clinical relevance. Given the high consanguinity rates in Egypt, the study’s focus on autosomal recessive inheritance patterns highlights the importance of genetic counseling and carrier screening in high-risk populations. Additionally, the integration of bioinformatics tools, such as I-Mutant 2.0, DynaMut2, mCSM and VarSome, for in silico pathogenicity prediction enhances the interpretation of novel and uncertain significance variants, providing valuable insights into their potential functional impact. The findings have direct clinical implications, reinforcing the role of early genetic diagnosis, personalized treatment strategies, and family screening in improving disease management and reproductive decision-making.

### Study limitations

Despite its strengths, this study has several limitations that warrant consideration. The sample size (26 patients) is relatively small, which may limit the generalizability of the findings and, importantly, precludes statistically meaningful enrichment analysis to determine whether the identified variants are more prevalent in the Egyptian population compared to global databases. Additionally, while targeted NGS panels provide high diagnostic accuracy for known disease-associated genes**,** they do not capture variants in genes outside the panel. WES or whole-genome sequencing (WGS) could provide a broader genetic landscape and uncover additional causative mutations.

Sanger sequencing and segregation analysis were performed only for families that completed clinical follow-up. Although variant calling was conducted using a high-confidence NGS pipeline with rigorous alignment and filtering to minimize pseudogene interference, orthogonal validation (e.g., through MLPA or qPCR) was not performed, warranting cautious interpretation of some findings.

Furthermore, functional validation of novel variants was not undertaken; pathogenicity assessments relied primarily on in silico prediction tools without supporting evidence from experimental studies. Moreover, while benign variants were initially detected, these were filtered out during the GATK pipeline analysis, which may occasionally risk excluding variants of uncertain or modifier effect. Additionally, confirmatory Sanger sequencing could not be performed in some cases due to limited DNA availability and patient follow-up, as illustrated by P22. This limited our ability to resolve ambiguous zygosity calls and represents a key methodological limitation.

Accordingly, variants initially classified as VUS were not reclassified as pathogenic or likely pathogenic unless additional supporting evidence was available, in line with ACMG guidelines. Structural variants and copy number variations (CNVs), which can play a significant role in inherited kidney diseases (IKDs), were also not assessed. Additionally, although consanguinity status was reported, formal quantification of the inbreeding coefficient (F) for each proband was not performed, which limits deeper genetic correlation analyses and should be considered in future studies. Lastly, referral bias may have influenced the variant spectrum observed, as patients were selected based on clinical suspicion of IKDs.

## Conclusion

This study provides comprehensive genetic insights into inherited kidney diseases (IKDs) among Egyptian patients, highlighting 17 distinct pathogenic, likely pathogenic, and VUS variants across 12 genes, including six novel variants. Through targeted NGS, coupled with rigorous pipeline filtering (which excluded benign variants), segregation analysis, and in silico functional prediction tools, we identified disease-causing mutations associated with AS, CAKUT, renal tubular dysgenesis, and PKD, reinforcing the critical role of genetic screening in early diagnosis and clinical management of IKDs.

Among the most significant findings, *COL4A3* and *COL4A5* mutations were the most frequently detected, underscoring Alport Syndrome as the predominant genetic kidney disorder in this cohort. The identification of a novel *COL4A3* variant (c.3926C > A) predicted to affect protein stability and classified as a VUS, in the context of a previously reported pathogenic alteration at the same codon, suggests a key contribution of collagen IV gene mutations to renal dysfunction in the Egyptian population.

Additionally, novel variants in *FREM1* and *AGT* were detected and supported by structural destabilization and ACMG criteria, indicating a potential previously unrecognized role of these genes in syndromic and developmental renal disorders. The presence of *FREM1* mutations in multiple patients suggests its strong involvement in CAKUT-related syndromes, while *AGT* and *ACE* mutations highlight the importance of the renin–angiotensin–aldosterone system (RAAS) in kidney development and blood pressure regulation.

The study also identified pathogenic mutations in *PKD1* and *PKHD1*, responsible for autosomal dominant and autosomal recessive polycystic kidney disease, respectively, demonstrating the utility of NGS in resolving genetically complex regions and improving diagnostic yield. These findings emphasize the genetic heterogeneity of polycystic kidney disorders and the importance of genetic screening for precise diagnosis and management.

Collectively, our results contribute to the growing body of evidence supporting precision nephrology in low- and middle-income countries. Implementing NGS-based genetic screening in clinical workflows can facilitate personalized care, risk stratification, and family counseling, particularly in populations with high consanguinity and early-onset kidney disease [70].

## Supplementary Information

Supplementary Material 1. 

## Data Availability

The data presented in this study are available on request from the corresponding author.
