# Supplementary material for: Genetic etiology of inherited kidney diseases in egyptian patients: next generation sequencing identifies six novel variants
Source: Mol Cell Pediatr. 2025 Nov 25;12:22. doi: 10.1186/s40348-025-00203-2 (PMC12647436; doi:10.1186/s40348-025-00203-2)
Supplement: Supplementary file 1 — Supplementary Material 1. [file 40348_2025_203_MOESM1_ESM.docx]

**Supplementary File**

**Table S1: Kidney Disease Gene Panel – Genes and Transcripts**

| **Gene Symbol** | **Reference Transcript (RefSeq)** | **Associated Disease(s)** |
| --- | --- | --- |
| ***COL4A3*** | NM_000091.5 | Alport syndrome, TBMN |
| ***COL4A5*** | NM_033380.3 | Alport syndrome (X-linked) |
| ***FREM1*** | NM_144966.7 | Bifid nose with or without anorectal and renal anomalies |
| ***ACE*** | NM_000789.5 | Renal tubular dysgenesis |
| ***AGT*** | NM_000029.4 | Renal tubular dysgenesis |
| ***PKD1*** | NM_001009944.2 | ADPKD |
| ***PKHD1*** | NM_138694.3 | ARPKD |
| ***NPHS2*** | NM_014625.4 | SRNS, FSGS |
| ***TBC1D8B*** | NM_017752.3 | Nephrotic syndrome |
| ***NOTCH2*** | NM_024408.3 | Alagille syndrome, CAKUT |
| ***BBS4*** | NM_033028.3 | Bardet-Biedl syndrome |
| ***CYP24A1*** | NM_000782.5 | Infantile hypercalcemia |
| ***WT1*** | NM_024426.6 | Denys-Drash, Frasier syndrome |
| ***LAMB2*** | NM_002292.3 | Pierson syndrome |
| ***INF2*** | NM_022489.4 | FSGS, CMT syndrome |
| ***TRPC6*** | NM_004621.6 | FSGS (autosomal dominant) |
| ***HNF1B*** | NM_000458.4 | RCAD syndrome |
| ***PAX2*** | NM_003987.3 | Renal coloboma syndrome |
| ***CLCN5*** | NM_000084.4 | Dent disease (X-linked) |
| ***SLC34A1*** | NM_001177316.1 | Nephrolithiasis, Fanconi |
| ***COQ6*** | NM_182480.4 | CoQ10 deficiency, SRNS |
| ***ADCK4*** | NM_032514.4 | CoQ10-related nephropathy |
| ***NPHS1*** | NM_004646.4 | Congenital nephrotic syndrome |
| ***UMOD*** | NM_003361.3 | AD tubulointerstitial disease |

***Abbreviations: ***

COL4A5: Collagen type IV alpha 5 chain, COL4A3: Collagen type IV alpha 3 chain, FREM1: FRAS1-related extracellular matrix 1, AGT: Angiotensinogen, PKD1: Polycystin 1, PKHD1: Polycystic kidney and hepatic disease 1, NPHS2: NPHS2 stomatin family member, podocin, TBC1D8B: TBC1 domain family member 8B. NOTCH2: Notch receptor 2, BBS4: Bardet Biedl syndrome 4, CYP24A1: Cytochrome P450 family 24 subfamily A member 1.ADPKD – Autosomal Dominant Polycystic Kidney Disease, ARPKD – Autosomal Recessive Polycystic Kidney Disease, TBMN – Thin Basement Membrane Nephropathy, SRNS – Steroid-Resistant Nephrotic Syndrome, FSGS – Focal Segmental Glomerulosclerosis, CMT – Charcot-Marie-Tooth disease, CAKUT – Congenital Anomalies of the Kidney and Urinary Tract, RCAD – Renal Cysts and Diabetes syndrome, AD – Autosomal Dominant, X-linked – X-linked inheritance

**
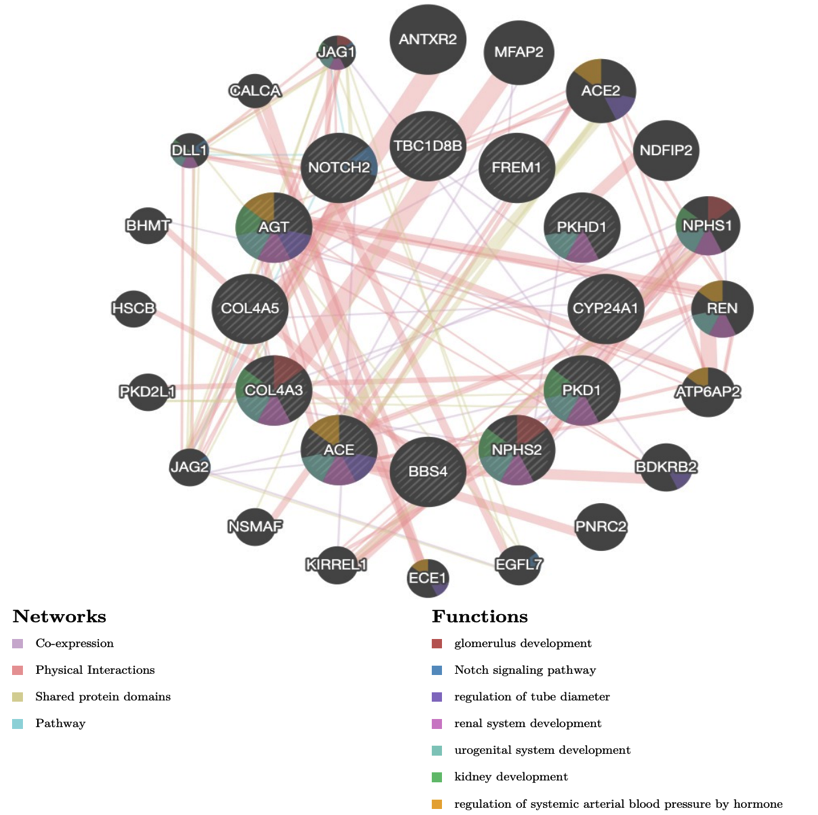
Figure S1: geneMANIA plot shows the interaction between the targeted genes.** The interactions between proteins were presented in terms of co-expression, physical interaction, shared protein domains, and common pathways with different related functions.
